# Unliganded Estrogen Receptor Alpha Promotes PC12 Survival during Serum Starvation

**DOI:** 10.1371/journal.pone.0069081

**Published:** 2013-06-25

**Authors:** François Ferriere, Denis Habauzit, Farzad Pakdel, Christian Saligaut, Gilles Flouriot

**Affiliations:** Transcription, Environment and Cancer Group, Institut de Recherche sur la Santé, Environnement et Travail (IRSET), Institut National de la Santé et de la Recherche Médicale (INSERM) U1085, Université de Rennes 1, Rennes, France; Institut de Génomique Fonctionnelle de Lyon, France

## Abstract

Many studies have reported proliferative, differentiating or protective effects of estradiol, notably through estrogen receptor alpha (ERα). On the contrary, the ligand-independent action of ERα is currently poorly documented notably in cell protection. The stable transfection of wild type, substituted or truncated form of ERα in PC12 cells (ERα negative cell line) lead the specific study of its ligand-independent action. Hence, we demonstrate here that, in the absence of E_2_, the expression of ERα prevents cells from apoptosis induced by serum deprivation. This protection is not due to an ERE-mediated transcription and does not require either AF-1 or AF-2 transactivation functions. It is afforded to the Y537 residue of ERα and activation of c-Src/Stat3 signaling pathway.

## Introduction

17β estradiol (E_2_) exerts crucial influences in growth, differentiation and homeostasis of different tissues in both females and males [1]. Many of these effects are mediated through binding and activation of intracellular receptors that belong to the nuclear receptor subfamily of ligand-inducible transcription factors, the estrogen receptors α (ERα) and β (ERβ). Among them, ERα is thought to mediate many of the trophic and protective effects of E_2_ [2–5]. Ligand-activated ERα undergoes a conformational change that facilitates its recruitment onto the promoter regions of target genes [6]. This recruitment is either direct through interaction with consensus DNA sequences (ERE, Estrogen Responsive Elements), or through protein/protein interaction with other transcriptional factors such as Sp1 or AP-1. In addition to its nuclear action, ERα also mediates rapid membrane-initiated actions of E_2_: changes in adenyl cyclase, mitogen-activated protein kinases (MAPKs), phosphatidylinositol 3-kinase (PI_3_K) activities and modulation of intracellular calcium concentration [7]. Although most ERα proteins reside in the nucleus, a population of ERα molecules is localized to the cytosolic and membrane compartments, either through post-translational modifications and/or through interaction with membrane proteins [8–12]. Although these rapid actions of E_2_ are mainly characterized by processes affecting some signal transduction pathways, the subsequent cellular response may ultimately be a regulation of gene expression [13,14]. ERα can also act in a ligand-independent manner. Several studies reported an activation of the unliganded ERα by phosphorylation [15,16]. Unliganded ERα can also find access to the heterochromatic promoter in order to initiate events leading to chromatin remodeling and transcription initiation [17]. We previously demonstrated that unliganded ERα exhibites a negative influence on NGF-induced neuritogenesis in PC12 cell line, using non-transcriptional mechanism [5]. Moreover, PC12 cell line rapidly undergoes cell death following serum and NGF withdrawal or following some oxidative shocks and ERα-positive PC12 cells are significantly more resistant in serum-free conditions than control ones, even in the absence of estrogen treatment [18]. Nevertheless, the precise mechanism by which ERα exerts its influences on cellular protection is still pending. In this study, we aimed to identify the mechanism of protection afforded to unliganded ERα in PC12 cells subjected to serum deprivation. Our PC12 cell line does not express endogenous ERα in its native state. That allows to determine the mechanisms of possible transcriptional or membrane-initiated effects of ERα on cell apoptosis and survival by the use of PC12 clones, resulting of a stable transfection with different mutated or deleted forms of ERα [4,5,19].

## Materials and Methods

### Materials

E_2_ and ICI 182,780 (ICI) were respectively purchased from Sigma Aldrich (St Louis, MO, USA) and from Tocris (Bristol, UK). STAT3 inhibitor V, Stattic (InhV) was purchased from Merck. PP2 (4-amino-5-(4-chlorophenyl)-7-(t-butyl) pyrazolol[3,4-d] pyrimidine), LY294002 (2-(4-Morpholinyl)-8-phenyl-4H-1-benzopyran-4-one) and PD98059 (2’-amino-3’-methoxyflavone) were obtained from Invitrogen. Stat3(1)-luciferase-reporter vector was purchased from Ozyme. Antibodies against Erk_1-2_ (K-23), ERα (HC20) and Src (B12) were purchased from Santa Cruz Biotechnology (California, USA). The anti-nonP(Y527)-Src, anti-P (Y416)-Src and anti-P (Y705)-Stat3 antibodies were from Upstate (New-York, USA).

### Cell culture

The PC12 cell line was derived from rat pheochromocytoma, a tumor arising of the adrenal medulla [20] and represents a valuable model to study cell fate such as neuronal differentiation, cell proliferation, cell survival or differentiation [4,5,18,21]. PC12 cells were grown in phenol red-free Dulbecco’s modified Eagle’s medium with F12 (DMEM/F12; Sigma-Aldrich) supplemented with 8% of charcoal-stripped fetal calf serum (FCS, BioWest), 2% of charcoal-stripped horse serum (HS; Sigma-Aldrich), 1% of a solution containing 10 µg/ml penicillin G, 10 mg/ml streptomycin, and 25 µg/ml amphotericin B in 0.9% NaCl, 1 mM pyruvate-sodium and 4 mM L-glutamine (Sigma-Aldrich).

All stable transfected PC12 cells were derived from PC12 wild type (PC12-WT). Stably transfected PC12 clones (PC12-C2, PC12-ER1, PC12-ER2, PC12-ER-ΔA, PC12-ER-ΔA/Box1, PC12-ER-CF, PC12-ER-DF, PC12-ER-ΔAF-2, PC12-ER-Δc-term and PC12-ER-Y537S) were obtained in our lab as previously described [4,5] following stable transfection of PC12 cells with the corresponding expression vectors using FuGENE 6 reagent (Roche Diagnostics, Bâle, Switzerland), and selection with 0.8 mg/ml G418 (Invitrogen, Carlsbad, USA). Expression vectors for ERα, ERα-ΔA (amino acids 39-595), ERα-CF (amino acids 174-595), ERα-ΔC-term (amino acids 1-532) cDNAs have been already described [4,5]. ERα-ΔA/Box 1 (amino acids 80-595; Dr. F. Gannon, EMBL, Germany), cDNAs are gifts. The cDNAs encoding ERα-DF (amino acids 256-595), ERα-Y537S [22] and ERα-∆AF-2 were obtained by PCR using ERα cDNA as template. Primers sequences are available upon request. All cDNAs were subcloned into the expression vector pCR3.1, with the exception of ERα-DF cDNA which was subcloned into pCDNA3.1 TOPO (Invitrogen, Carlsbad, USA).

The MCF7 human breast cancer cell lines were purchased from the American Type Culture Collection (Manassas, VA, USA). MCF7 cells were routinely maintained in DMEM (Invitrogen, Cergy potoise, France) supplemented with 10% of FCS (Biowest) and antibiotics (Sigma-Aldrich) at 37°C in 5% CO_2_.

### Serum starvation and assessment of cell viability

Cells were plated onto multi-well plates at 4x10^4^ cells per well in phenol red-free DMEM/F12 containing 5% of charcoal stripped FCS (Sigma-Aldrich). Twenty four hours later, cells were transferred into phenol red-free DMEM/F12 containing either 5% of charcoal stripped FCS (Sigma-Aldrich) or 0% of FCS for 24 hours, in the presence or in the absence of inhibitors (PP2, InhV, PD98059 and LY294002) and treated with vehicle control, E_2_ or ICI. Cell viability was then assessed by quantification of cellular ATP content (ViaLight HS kit, Lonza).

For siRNA experiments, 10^4^ MCF7 cells per well were seeded in 96-well plates one day before transfection, in phenol red-free medium. The cells were then transfected in triplicate with siRNA targeting human ERα (Santa Cruz Biotech), using Lipofectamine 2000 (Invitrogen) according to the manufacturer’s instructions. Cells transfected with nonspecific siRNA (Santa Cruz Biotech) were used as controls. One day after transfection, the MCF7 cells were cultured in 100 µL of phenol red-free DMEM/F12 containing either 10% of charcoal stripped FCS (Sigma-Aldrich) or 0% of FCS for 24 hours before cell viability was assessed using ViaLight HS kit (Lonza).

### Caspase-3 activity assays

Control PC12 cells or serum-starved PC12 cells for 24 hours were centrifuged, washed with PBS and suspended in 80 µl of extraction buffer (25 mM d’HEPES (pH 7.5), 5 mM MgCl_2_, 5 mM EDTA, 5 mM DTT, sucrose 10%, CHAPS 0.1%) and incubated 10 min on ice. After centrifugation (15 min at 15,000 g), the synthetic fluorescent peptide Ac-DEVD-AMC (100 µM; Biomol) was added to the supernatant in the reaction buffer (20 mM HEPES (pH 7.5), 100 mM NaCl, 1 mM EDTA, 10 mM DTT, sucrose 10%, CHAPS 0.1%). Fluorescence was measured at 380 nm for excitation and 460 nm for emission (Fluorolite 1000, Dynatech). Enzyme activity was expressed as fluorescence units per minute per mg protein. The protein concentration was determined by the Bradford method.

### Transient transfection experiments

For luciferase and β-galactosidase assays, transient transfections were performed with FuGENE 6 transfection reagent (Roche Diagnostics), as recommended by the manufacturer, in 24-wells plates. Total DNA mixture included 200 ng of reporter genes (Stat3(1) luciferase reporter vector or ERE-tk-luciferase), 150 ng of CMV-βGal internal control. After 36 hours of transient transfection, cells were harvested and luciferase and β-galactosidase assays were performed as previously described [23]. The reporter gene activity was obtained after normalization of the luciferase activity with the β-galactosidase activity.

### Western blot analysis

Forty µg of whole cell extracts were denatured in a Laemmli buffer at 95°C for 5 min, resolved on a 10% SDS-PAGE, and electrotransferred onto PVDF membranes (Amersham Biosciences). After blocking, membranes were incubated with the primary antibody in 5% milk/0.1% Tween/PBS for 1.5 hours at room temperature. After washings, blots were incubated with appropriate secondary peroxidase-conjugated antibody for 1 hour. Membrane-bound secondary antibodies were detected using the ECL (extra chemiluminescence) from Amersham Biosciences. Protein level ratios were analysed by densitometric analyses using the ImageJ software.

### ER and Src sub-cellular localization by immunocytochemistry

MCF7 cells, PC12-C2, PC12-ER2 and PC12-ER-Y537S (7x10^4^ cells/ml) were grown on coverslips into 24-well plates in phenol red-free DMEM/F12 containing 5% of charcoal stripped FCS and were treated or not with E_2_ (10 nM) for 5 min. Cells were fixed in 4% paraformaldehyde for 10 min, washed in large amount of PBS. Cells were then saturated and permeabilized during 1 hour using PBS with 0.2% triton and 0.5% non-fat milk and were then incubated overnight at 4°C with the primary antibodies (1/1000) anti-ER (HC20) or anti-Src (B12). After washes, coverslips were incubated with respectively anti-rabbit or anti-mouse fluorescently labeled second antibodies (1/500) for 45 min at room temperature. The coverslips were then mounted with Mounting Medium containing Dapi and were analyzed on fluorescence microscope.

### Proximity Ligation Assay (PLA)

The Proximity Ligation Assay (PLA) technology allows visualization of protein/protein interactions *in situ*. Cells were grown as described for immunocytochemistry and the coverslips were treated according to manufacturer’s instructions (Duolink II Fluorescence, Olink Bioscience, Sweden [24]; commercialized by Sigma-Aldrich). Firstly, the samples were saturated and permeabilized 1 hour using PBS with 0.2% triton and 0.5% non-fat milk. Then, couple of primary antibodies (HC20, rabbit anti-ER and mouse anti-Src, 1/1000 each) was incubated overnight at 4°C in PBS with 0.2% triton and 0.5% non-fat milk. After washes, the PLA minus and plus probes (containing the secondary antibodies conjugated with complementary oligonucleotides) were added and incubated 1 hour at 37°C. The next step allows the ligation of oligonucleotides if the two proteins are in close proximity thanks to the ligase during an incubation of 30 min at 37°C. After washes, the addition of nucleotides and polymerase and incubation of 100 min at 37°C allows a rolling-circle amplification reaction using the ligated circle as a template. The amplification solution also contains fluorescently labeled oligonucleotides that hybridize to the rolling-circle amplification product. The coverslips are let drying at room temperature in the dark, mounted with Duolink II Mounting Medium containing Dapi and analyzed on fluorescence microscope (Zeiss AxioImager Z1 microscope with objective X20). On each sample, at least 500 cells were counted. Analyses and quantifications of these samples were performed using ImageJ software that allows counting dots on 8 bits image and the plugin “Counter cells” allows analyzing cells number.

### Statistical analysis

The statistical analyses were performed by Student t test. Interactions between variables (pharmacological treatments, expression of ER) were performed using the analysis of variance (ANOVA) followed by analysis of individual group differences using the Statview 5.0 software (SAS Institute Inc., Cary, NC).

## Results

### Unliganded ERα protects PC12 cell against apoptosis

**Figure 1:**
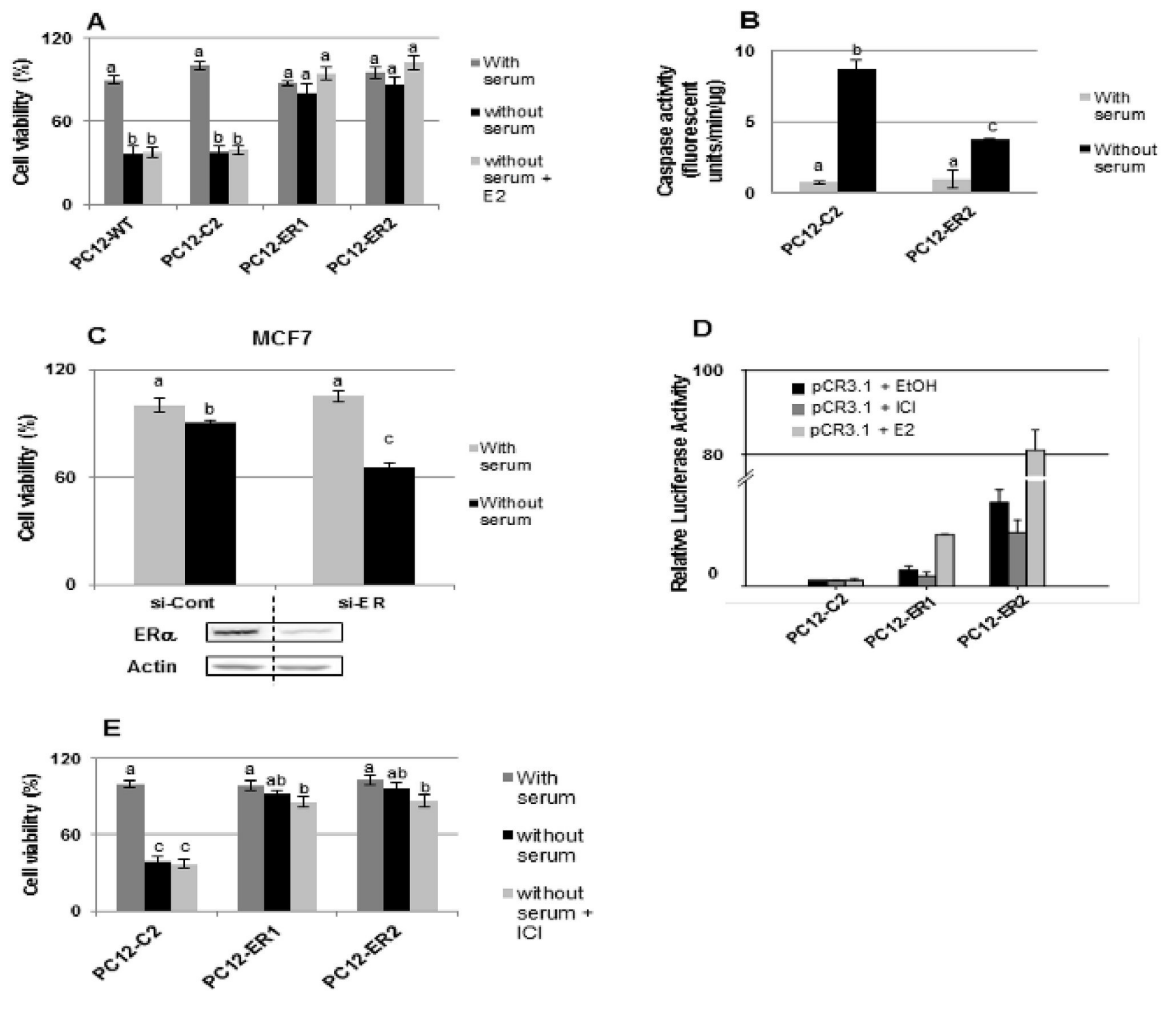
Unliganded ERα protects cells against apoptosis. A) - PC12 wild type cells (PC12-WT), PC12 cells transfected with the empty plasmid (PC12-C2) or PC12 stably transfected with ERα encoding plasmid (clones PC12-ER1 and PC12-ER2) were grown in the presence or not of charcoal stripped FCS for 24 hrs with or without E_2_ (10^-8^ M). Cell viability was then assessed using the quantification of cellular ATP (Vialight HS kit from Lonza). Results are expressed in reference with control clone (PC12-C2) maintained in 5% charcoal stripped FCS. B) - caspase-3 activity was determined in control clone (PC12-C2) and in ERα-positive cells (PC12-ER2) after 24 hrs of culture in the presence or not of charcoal stripped FCS. C) - MCF7 cells were transfected with siRNA targeting ERα or with non-specific siRNA. Twenty four hours later, MCF7 cells were maintained in phenol red-free DMEM/F12 medium containing or not 10% charcoal stripped FCS for 24 hrs before cell viability was assessed using ViaLight HS kit (Lonza). In order to control knockdown efficiency, total protein was extracted from MCF7 cells and the level of ERα was analyzed by western blotting. D) - PC12-C2, PC12-ER1 and PC12-ER2 cells were transiently transfected with an ERE-TK-Luc reporter gene together with CMV-β-Gal. Twelve hours after transfection, the cells were treated or not with E_2_ (10^-9^ M) or ICI (10^-7^ M) for 24 hrs. Normalized luciferase activities were standardized to the reporter gene activity measured in ethanol treated PC12-C2 cells. E) - PC12-C2, PC12-ER1 and PC12-ER2 cells were grown in medium with or without FCS for 24 hrs, in the presence or not of ICI (10^-7^ M). Cell viability was then assessed using ViaLight HS kit (Lonza). Results are expressed in reference with control clone (PC12-C2 maintained in 5% charcoal stripped FCS. For all the experiments, values correspond to the mean ± SEM of at least three separate experiments. Columns with different superscripts differ significantly (p<0.05 by Student test).

Serum deprivation for 24 hours was accompanied by a high mortality of the wild type PC12 cells (PC12-WT) or of PC12 cells transfected with the empty plasmid (PC12-C2) (Figure 1A). Interestingly, stable expression of ERα improved the cell viability in absence of estrogen, measured by Vialight HS kit from Lonza (Figure 1A), by trypan blue exclusion test or by flow cytometry analysis (data not shown) for two different PC12-ER clones (ER1 and ER2). Treatment with E_2_ for 24 hours during serum deprivation had no significant effect on cell viability (Figure 1A). The caspase-3 activity, as an apoptosis index, was dramatically decreased in PC12-ER cells when compared with control ones, after 24 hours of serum deprivation (Figure 1B). Thus, it seems that unliganded ERα confers PC12-ER cell protection against apoptosis. In order to ensure that this ligand-independent protection does not depend on the cell type, survival was studied in ERα-positive breast tumor cell MCF7 treated with specific siRNA directed against ERα. As for PC12 cells, MCF7 cells were maintained in phenol red-free medium supplemented with charcoal-stripped fetal calf serum to ensure that no steroids were present. Specific siRNA induced a reduction of nearly 70-80% of ERα expression compared with that of MCF7 control ones (Figure 1C). MCF7 cells transfected with the ERα siRNA were statistically less resistant to serum starvation than cells transfected with the control siRNA (Figure 1C). This result demonstrates that the protection afforded to unliganded ERα is not dependent on our cell model but could be reproduced in ERα-positive cells. In order to confirm that the protection in PC12 cells is ligand-independent, the ERα-dependent transcriptional activity was determined in the presence or not of estrogen and of ICI (Figure 1D). E_2_ increased luciferase activity in the PC12-ER (PC12-ER1 and PC12-ER2) but not in PC12-C2 cells suggesting that transfected ERα is a transcriptionally functional form. The basal transcriptional activity (i.e. in the absence of E_2_) was higher in PC12-ER2 than in PC12-ER1 (Figure 1D). This is in accordance with the higher expression of ERα in PC12-ER2 than in PC12-ER1. Interestingly, the antagonist ICI induced a light but not significant diminution of the basal transcriptional activity in PC12-ER2 or in PC12-ER1, probably due to the degradation of ERα induced by ICI treatment. The absence of residual estrogen in our culture medium, implicated in ER-induced protection, was further confirmed by the fact that ICI did not significantly decrease cell viability during serum deprivation (Figure 1E). These results confirm that a residual hormone is not present in the medium and that the protection induced by ER is actually ligand-independent.

### The ERα-dependent protection against serum deprivation is not mediated by an ERE-mediated transcription and by AF-1 and AF-2 transactivation functions

**Figure 2:**
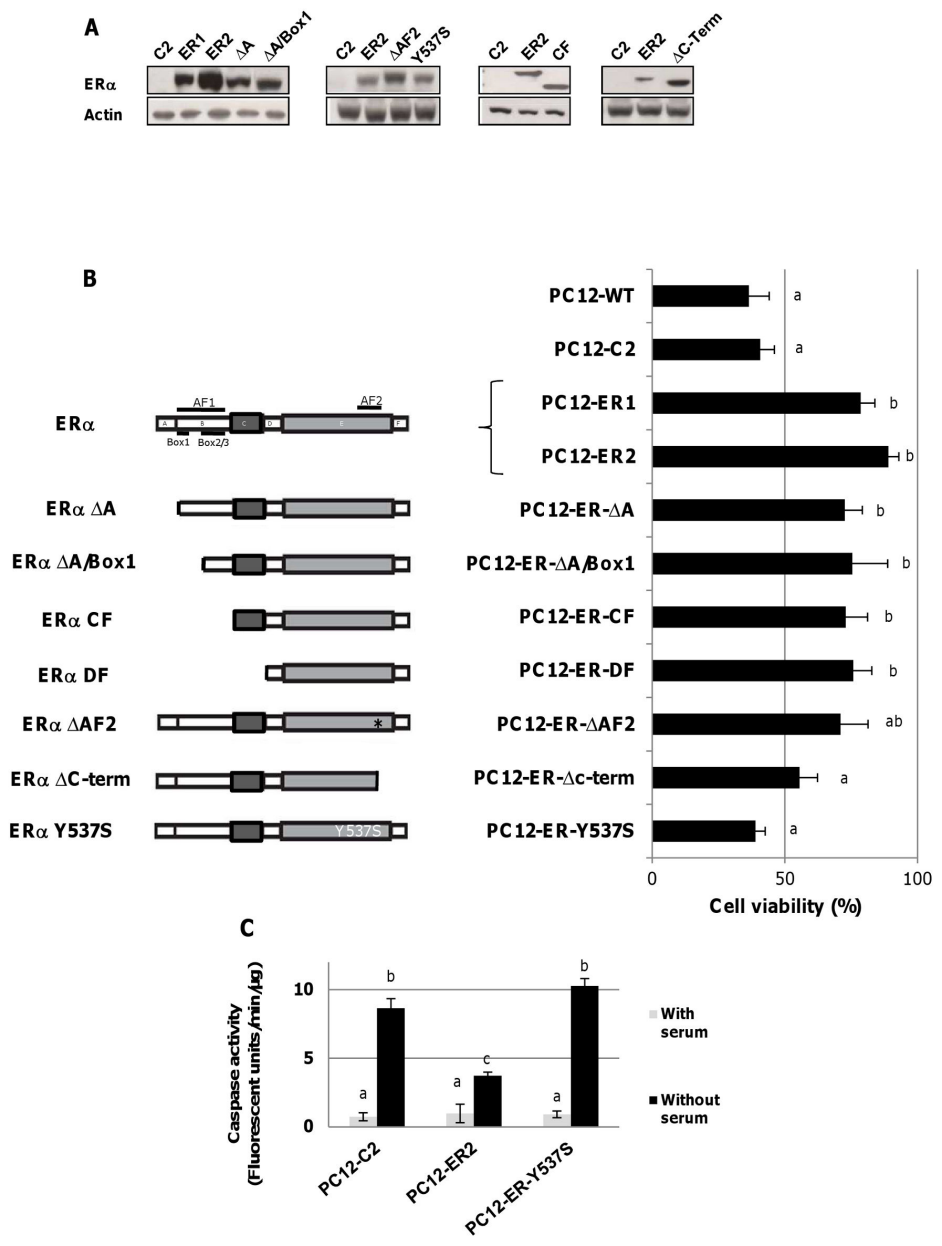
Ligand-independent protection against apoptosis depends on tyrosine 537 of ERα. A) - Total protein was extracted from different PC12 clones and the level of ERα was analyzed by Western blotting. B) - Cell viability (right panel) of mutated PC12 clones (left panel) was determined after 24 hrs of serum deprivation using ViaLight HS kit (Lonza). For each clone, viability was expressed in percent in reference to the viability of the same clone maintained in medium containing 5% charcoal stripped serum (value 100). Values correspond to the mean ± SEM of at least three separate experiments. Columns with different superscripts differ significantly (p<0.05 by Student test).

In order to identify the domains needed for the protective effects of unliganded ERα, PC12 clones stably expressing different mutated or deleted ERα deficient for classical functions were subjected to serum deprivation. As shown in Figure 2A, our PC12 individual clones were chosen in order to minimize the discrepancy in protein expression. The protective effect of ERα was still detectable in PC12-ER-ΔA clones (deleted of the A domain), in PC12-ER-ΔA/box 1 clones (deleted of the A domain and of the AF-1 sub-region box 1) and in PC12-ER-CF clones (lacking the entire A/B domain and deficient for the full AF-1 function) (Figure 2B). Likewise, the deletion of the ERα C-terminal region containing the helix 12 (ERα-ΔAF-2), which is a critical secondary structure for AF-2 transactivation efficiency, had no impact on the protective action of ERα. Finally, the protection still occurred in PC12-ER-DF cells, lacking the DNA Binding Domain (DBD) and then unable to bind DNA. Altogether these data suggest that the ligand-independent protection does not require AF-1 and AF-2 transactivation functions and that a direct transcription mediated by ERE is not implicated.

### The protective effect of unliganded ERα involves the tyrosine 537 of ERα and its implication in c-Src/Stat3 pathway

**Figure 3:**
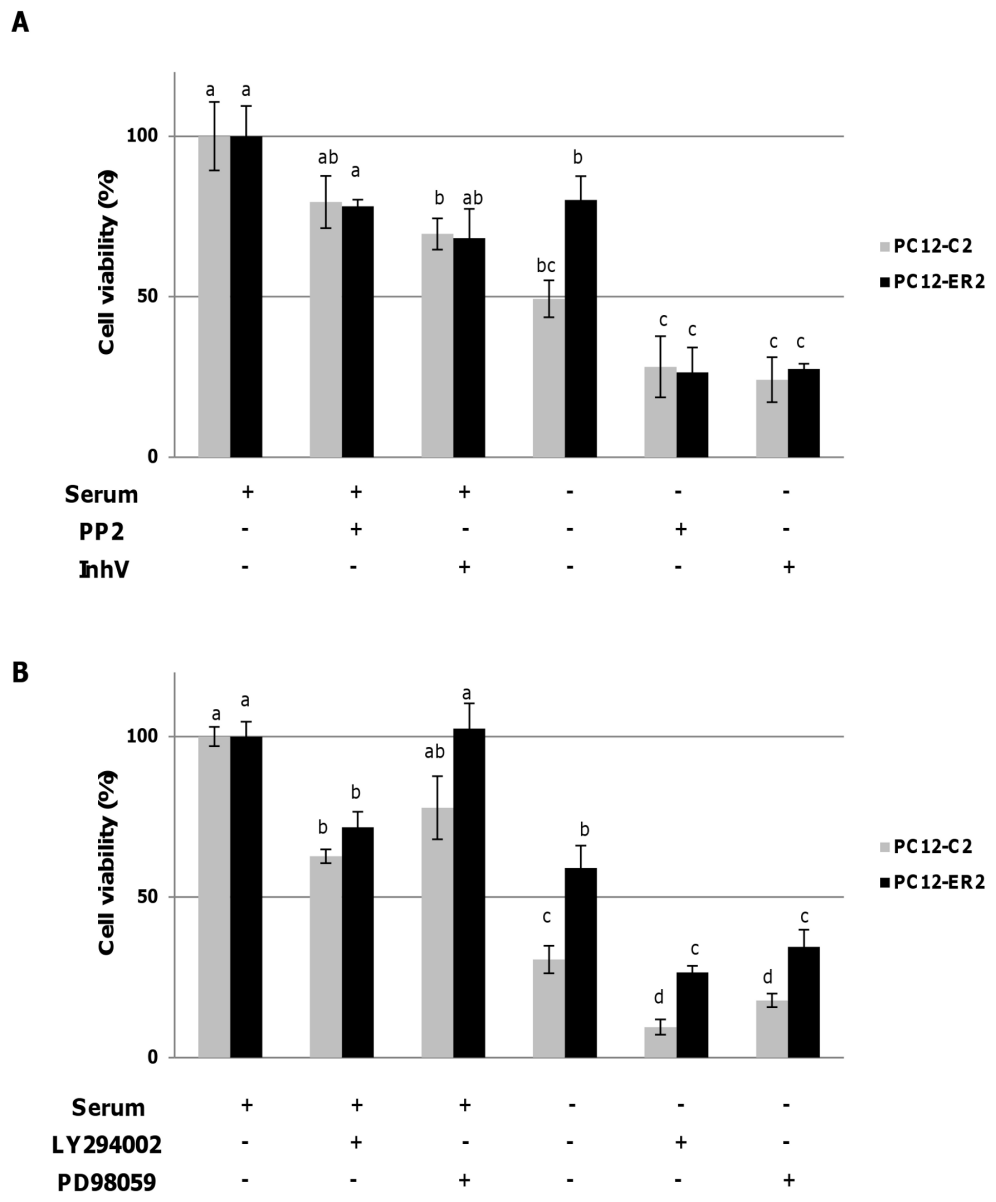
The protective effect of unliganded-ERα depends on c-Src and Stat3 activities. PC12-C2 and PC12-ER2 cells were grown in medium with or without FCS for 24 hrs, in the presence of inhibitors of c-Src or Stat3 (respectively PP2 and InhV, panel A) or inhibitors of PI3K/Akt or Mek/Erk (respectively LY294002 and PD98059, panel B). Cell viability was then assessed using ViaLight HS kit (Lonza). Results were expressed in cell viability in percent relatively to the viability of the same cells maintained in medium containing FCS without inhibitors. Values correspond to the mean ± SEM of three separate experiments. No significant interaction occurred following 2 way AOV between treatments with PP_2_ or InhV and ER expression. Columns with different superscripts differ significantly (p<0.05 by Student test).

Cells expressing ERα deleted for amino acids 533-595 (PC12-ER-Δc-term) are not protected against serum deprivation-induced apoptosis (Figure 2B). This deleted region contains the helix 12 of ERα, the end of E-domain and all the F-domain. The deleted E-domain possesses the tyrosine 537 (Y537) that interacts with the SH2 domain of c-Src [25], initiating numerous cell signaling pathways [26]. The substitution of Y537 by a serine in the clone PC12-ER-Y537S evidences a decrease in cell viability and a return of cell viability equivalent to control one (Figure 2B). This PC12-ER-Y537S clone expresses a mutated form of ERα where the substitution of tyrosine 537 by a serine prevents the interaction of ERα with c-Src [27]. Moreover, the caspase activities in these cells and control ones are similar (Figure 2C). Thus, the kinase c-Src seems to be implicated in the cellular protection against serum deprivation. To better target the signaling pathways involved in the protection of ERα PC12 cells, different kinase inhibitors were tested (Figure 3A,B): PP2, an inhibitor of tyrosine kinases Src family members, LY294002, a selective inhibitor of PI_3_K/Akt pathway, PD98059, an inhibitor selective MEK and inhV (Stat3-inhibitor-V), an inhibitor of Stat3 activation. As expected, inhibition of c-Src, Stat3, Akt or MEK key signaling pathways by PP2, InhV, LY294002 or PD98059 affects cell viability even in the presence of serum. However, these pharmacological treatments affect cell viability less than serum deprivation and its influence is not sufficient to mask the protective effects of ER. In these conditions, PC12-ER2 cells lose their capacity to be protected against serum starvation-induced apoptosis when treated with PP_2_ or InhV (significant interaction occurred following 2 ways AOV between treatment with PP_2_ or InhV and ER expression; Figure 3A) but not by treatment with LY294002 or PD98059 (Figure 3B). Our data suggest then that ER-induced protection in PC12 cells is mediated by Src kinase and activation of the transcription factor Stat3, as reported [28,29].

**Figure 4:**
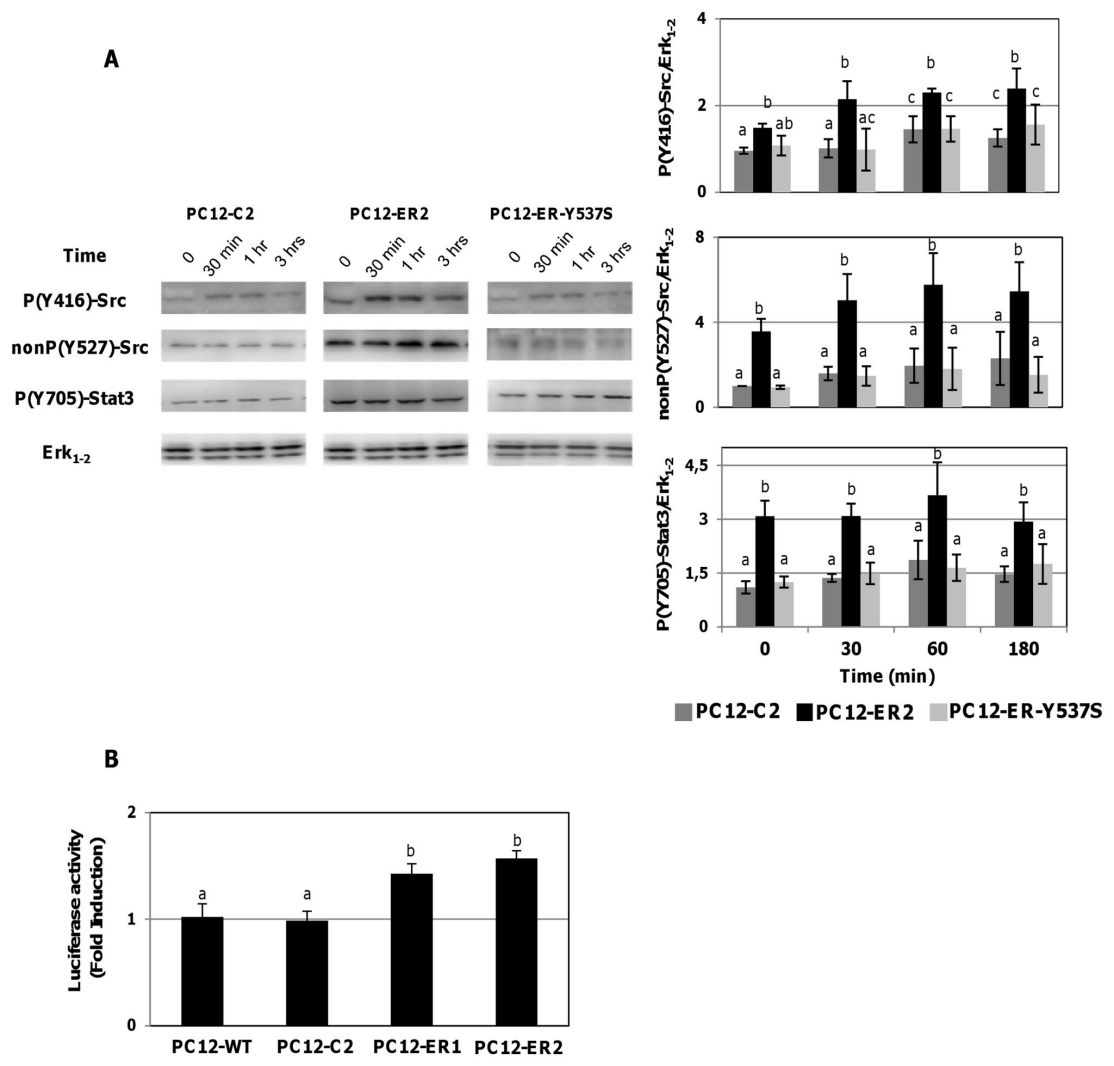
Unliganded-ERα increases Stat3 pathway activity. A) - The c-Src phosphorylation state (on tyrosine 416 and 527) and Stat3 (on tyrosine 705) were monitored during 3 hrs of serum deprivation by Western blotting in PC12-C2, PC12-ER2 and PC12-ER-Y537S cells. Total Erk_1-2_ was used as loading control. Histograms are the mean ± SEM of three separate experiments. In each case, results were expressed as the ratio of phosphorylation state/Erk_1-2_ for Src and Stat3. Columns with different superscripts differ significantly (p<0.05 by Student test). B) - Basal Stat3 transcriptional activity was determined in PC12-WT, PC12-C2, PC12-ER (ER1 and ER2) clones after 36 hrs of transient transfection. The Stat3 reporter gene activity was obtained after normalization of the luciferase activity with the β-galactosidase activity. Results correspond to the mean ± SEM of three separate experiments. Columns with different superscripts differ significantly (p<0.05 by Student test).

Two phosphorylation sites of c-Src have an opposite impact on the activity of the kinase. Phosphorylation of tyrosine 416, located in the catalytic domain, increases c-Src activity whereas phosphorylation of tyrosine 527, located in the C-terminal, decreases it by causing a repressive protein folding [30]. Hence, we studied the effects of serum deprivation on the phosphorylation status of tyrosine 416 and tyrosine 527 of c-Src in three cell lines: PC12-C2, PC12-ER2 and PC12-ER-Y537S. ER expression in PC12 cells increases the phosphorylation of tyrosine 416 in the presence of serum or during serum starvation (Figure 4A). In a same manner, ER expression in PC12 cells decreases the phosphorylation of tyrosine 527 in the presence of serum or during serum deprivation (increased of non-phosphorylated form). ER-induced protection may be related to simultaneous decreased phosphorylation of Tyr 527 of c-Src, increased the phosphorylation of Tyr 416 of c-Src and subsequent increased c-Src activity. ER cannot modulate the phosphorylation of both tyrosines 416 and 527 in PC12-ER-Y537S cells because ERα-Y537S cannot interact with c-Src. Therefore, c-Src phosphorylation states of Tyr 527 and Tyr 416 are similar in PC12-C2 and in PC12-ER-Y537S cells (Figure 4A). c-Src is known to increase the Stat3 tyrosine 705 phosphorylation. In this way, the activation of c-Src by ER increases downstream phosphorylation of Stat3 in PC12-ER cells but not in PC12-ER-Y537S, in the presence of serum or during serum deprivation. Therefore, the transcriptional activity of a Stat3 reporter vector is statistically higher in ER-positive cells compared to control or naïve cells (Figure 4B).

### The protection afforded to ERα is based on a ligand-independent interaction between ERα and Src

**Figure 5:**
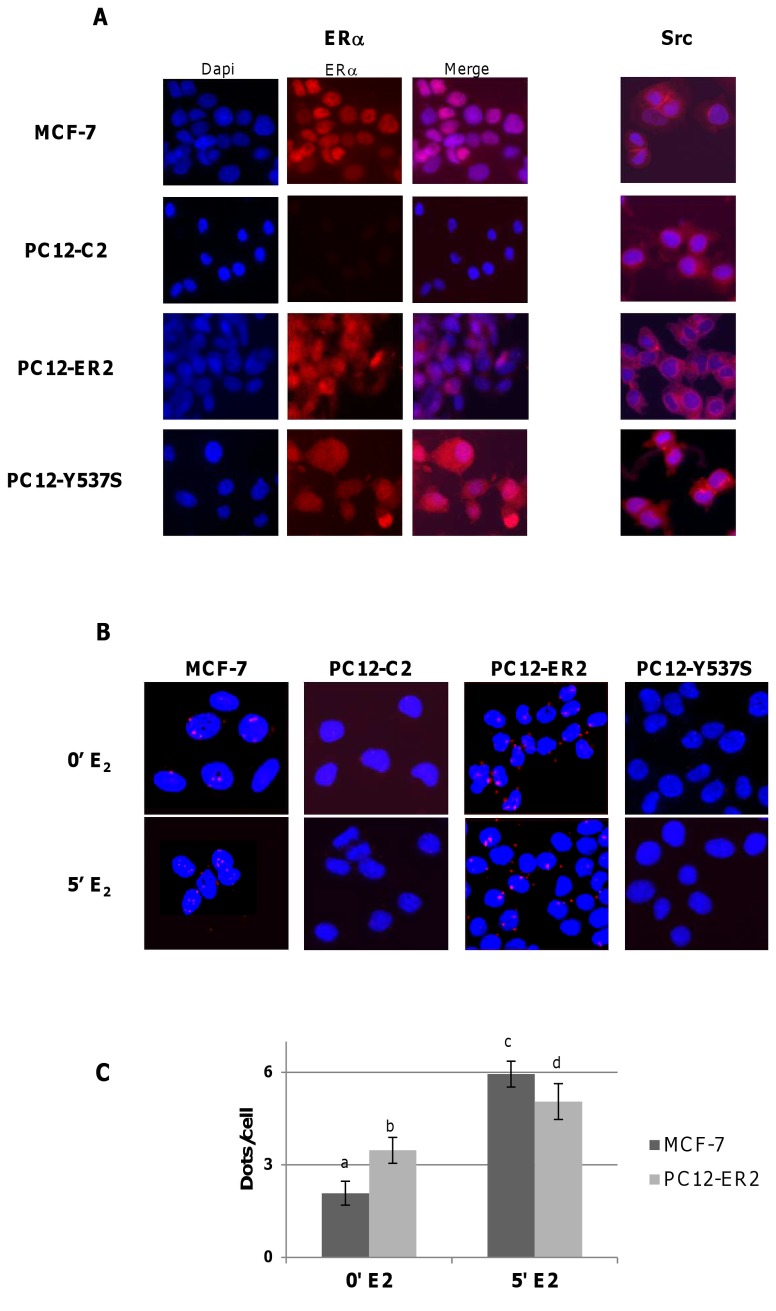
*In situ* PLA detection of endogenous ERα/Src interaction in PC12 and MCF7 cells. A) - Immunocytochemistry for ERα (left panels) and Src (right panels) in MCF7, PC12-C2, PC12-ER2 and PC12-ER-Y537S, grown in medium containing 5% of charcoal stripped serum. The nuclei were counterstained with Dapi (blue) (Obj: X20). B) - *In situ* PLA for ERα/Src dimers in MCF7, PC12-C2, PC12-ER2 and PC12-ER-Y537S grown in medium containing 5% of charcoal stripped serum with vehicle (upper panels) or with E_2_ (10^-8^ M) for 5 min (lower panels). The detected dimers are represented by red dots and the nuclei were counterstained with Dapi (blue). C) - Quantification of PLA signals per cell was performed by computer-assisted analysis as reported in the Materials and Methods section. Values correspond to the mean ± SEM of at least three separate experiments (10 fields; more than 500 cells/experiment). Columns with different superscripts differ significantly (p<0.05 by Student test).

Figure 5B shows that ERα interacted with Src in the absence of E_2_ in the cytoplasm of PC12-ER2 cells, as indicated by the presence of the red dots. Such an interaction was also observed in MCF7 cells (Figure 5B) and, as expected, no dots were detected in ER-negative PC12 cells (PC12-C2 cells; Figure 5B; not detectable in Figure 5C) or when we used only one of the antibodies (data not shown). The quantification of dots per cell indicated that this ERα/Src association is higher in PC12-ER2 cells than in MCF7 cells in the absence of E_2_ (Figure 5B,C). Interestingly, ERα was not confined to the nucleus in PC12-ER2 cells as a signal was detected by immunocytochemistry in the cytoplasm of these cells unlike in MCF7 cells where the signal was essentially nuclear (Figure 5A, left panels). The ER/Src interaction was enhanced by a short estrogenic treatment (5 min), especially in MCF7 cells (increased respectively 43% and 200% for PC12-ER2 and MCF7 cells; Figure 5B,C). Interestingly, no dots were detected in PC12-Y537S in the presence or in the absence of E_2_ (Figure 5B; not detectable in Figure 5C). These cells expressed ERα (Figure 5A) but the mutation in the tyrosine 537 abolished ER/Src interaction.

## Discussion

Our results demonstrate that ERα protects PC12 cells from apoptosis induced by serum deprivation in a ligand-independent manner. This ligand-independent protection is quite original since it is usually described in the literature as a ligand-dependent process. Nevertheless, Gollapudi and Oblinger [18] already reported a protection of ERα against serum withdrawal in stably transfected clone of PC12 cells. This increased survival was not related to an increased cell proliferation as the authors did not observe DNA replication when PC12-ER and PC12-control cells were moved into serum-free media conditions [18]. They suggested that the protection was due to the presence of exogenous serum steroids in the medium rather than a protective effect due to the mere expression of ERα. In the present study, we used a commercial serum which is stringently charcoal-stripped. Thus, in PC12 cells maintained in our steroid-free serum, the transactivation efficiency of ERα on an ERE-driven reporter gene (ERE-tk-Luc) was very low in the absence of any ligand and the treatment with ICI had no significant effect (Figure 1D). Therefore, the protection observed is actually ligand-independent.

This protection does not seem to be a cell-specific mechanism as we demonstrated a leading role of ERα in cell protection in MCF7 cells. While these cells are quite resistant to serum starvation in the absence of E_2_, they die en masse when the expression of ERα is decreased (Figure 1E). Finally, this ligand-independent protection is not specific to the paradigm used to induce apoptosis as we observed it when PC12-ERα cells were treated with staurosporine (data not shown). Such a ligand-independent protection afforded to ERα was recently described in human neuroblastoma cells where ERα expressing cells were more resistant against hydrogen peroxide-induced oxidative stress and staurosporine-induced apoptosis [31]. ER was the first steroid receptor to appear during evolution and our results strengthen the hypothesis that it might have initially been a transcription factor controlled by other signaling molecules than estrogens. It still retained some of its original functions, influencing by the way some cellular behaviors independently of its cognate ligand, estrogens, for which its sensitivity may have evolved later [32,33].

Our results clearly indicate that the transactivation functions (AF-1 and AF-2) and the DBD are not necessary for ERα to exert its protective effect. The AF-2-independent action is not surprising, as AF-2 is classically activated after ligand binding. If the AF-1 function is known to be active in the absence of ligand [34], it is not involved in PC12 protection as it is still detectable in clones deleted for the full AF-1 function. Moreover the ERα-Y537S receptor has been demonstrated to be constitutively transcriptionally active through recruitment of coactivators even in the absence of ligand [22,35]. Because PC12 cells stably expressing this ERα mutant show a similar behavior than control cells during serum deprivation, the role of direct transcriptional mechanisms in protection can be discarded. Then, the role of ERα membrane-initiated mechanisms can be evoked.

In such an hypothesis, ERα membrane-initiated mechanisms could involve the membrane-anchored tyrosine kinase c-Src [27]. The association of the phosphorylated tyrosine residue in position 537 of ERα with the SH2 domain of Src [25,36,37] is the essential starting point of a complex signaling network including Erk, phosphoinositide 3-kinase and various effectors of this kinase, such as Akt/PKB, Rac, and PKC [38]. Although an interaction between ER and Src is evidenced in the presence of E_2_ or growth factors, it has also been reported, albeit tenuous, in the absence of ligand [37–39]. Using PLA technology, we demonstrated that ERα and Src interact *in situ* in PC12-ER2 cells and also in MCF7 cells in the absence of E_2_. The complex ERα/Src was absent in PC12-C2 and in PC12-ER-Y537S where substitution of the tyrosine residue in position 537 with a serine is known to prevent the interaction of ERα and Src. Interestingly, the precise quantification of these *in situ* interactions demonstrated that this association is higher in PC12-ERα cells than in MCF7, in the absence of the ligand. This difference could be due to difference in the localization of ERα or difference of arginine methyltransferase PRMT1 activity between the two cell lines. Indeed, methylation of arginine 260 of ERα is required for mediating the extra-nuclear function of the receptor by triggering its interaction with Src and for propagating the signal to downstream transduction cascades that orchestrate cell proliferation and survival [40,41]. E_2_ increased ER/Src interaction but the effect was much lower in PC12-ER2 cells than in MCF7 cells. That could explain the poor protection induced by E_2_ in our experiments.

Src-dependent pathway has been involved on cell proliferation, cell apoptosis attenuation, cell differentiation, and angiogenesis [42–44]. C-Src plays a central role in many cellular functions involved in tumor progression and was the first proto-oncogene identified. In human cancers, the majority of Src dysregulation seems to occur *via* maintenance of an activated phosphorylated status. Moreover, a constitutive activation of Stat3 has been reported in a variety of tumor cells to depend upon c-Src [45] and should protect cells from apoptosis. Stat3 could also be involved in mechanisms of protection against heart and brain ischemia. Indeed, in animal models of transient focal ischemia, P-Stat3 co-localizes with the anti-apoptotic protein bcl-2, but not with cleaved-caspase-3 [46]. Activated Stat3 molecules could accumulate in the nucleus, where they induce transcription of many target genes, such as the one encoding Mcl-1, leading to anti-apoptotic effects [47]. These results are to be compared with our own observations: activating phosphorylation of tyrosine 416 of Src, due to ERα-Src interactions, is higher in PC12-ERα cells than in PC12-control ones. That increases P-Stat3 and its basal transcriptional activity, contributing to the ligand-independent protection afforded to ERα. Our results could explain the link between the expression of ERα and the down-regulation of main players modulating apoptosis (caspase-3, BAG3, BAG1) recently described in human neuroblastoma in absence of ligand [31].

This ligand independent activity of ERα could have significance in different physiological and physio-pathological contexts. In the case of tumor development, its organization implies that cells, especially those located in the center, are faced with an adverse physiological microenvironment (lack of nutriments, hypoxia…) which generally lead to apoptosis [48]. In ERα positive cells, the constitutive activation of c-Src/Stat3 pathway should confer protection against apoptosis.
