# Expression of Concern: Unliganded Estrogen Receptor Alpha Promotes PC12 Survival during Serum Starvation

**DOI:** 10.1371/journal.pone.0297382

**Published:** 2024-01-11

**Authors:** The PLOS ONE Editors

Following the publication of this article [1], concerns were raised regarding results presented in Figs 4 and 5. Specifically,

- In Fig 4A, the following panels appear similar despite being used to represent different conditions:
  ○ P(Y416)-Src: PC12-C2 and PC12-ER-Y537S
  ○ Erk_1-2_: PC12-C2 and PC12-ER2
- In the Fig 5B MCF-7 5’ E_2_ results, the background directly surrounding the cells appears to be different from the overall background of the panel.

Regarding the issues with Fig 4, the corresponding author stated that errors were made during figure preparation. They explained that not all data underlying the originally published Fig 4 are currently available, and commented that data files were provided to PLOS in 2015. The available underlying image data and some replacement panels presented in Figs 4A and 4B, as well as the individual-level data underlying the graphs presented in Figs 1–4, are provided in the S1–S3 Files available with this notice. PLOS is unable to access the journal’s 2015 correspondence records for this case. We sincerely regret that this case was not resolved much sooner after the prior correspondence.

Regarding the issues with Fig 5, the corresponding author stated that in order to improve the rendering of the figures after printing, they increased the contrast of areas containing cells of certain images to enable cell nuclei labelled with DAPI to be better defined and the red dots corresponding to interactions to be clearly seen in the illustrative panel B of Fig 5. The corresponding author was unable to clarify the origin of the Fig 5 concerns, and stated that the original images underlying this figure are no longer available. The authors provided a PowerPoint file (available in the S4 File with this notice) that, according to the corresponding author, represents Fig 5 as originally submitted to PLOS. In the absence of the underlying data, the issue with Fig 5 cannot be fully resolved, but the data provided in the S3 File do appear to support the published result.

The PowerPoint file linked to Fig 5 in the published article [1] appears to be corrupted.

The *PLOS ONE* Editors issue this Expression of Concern to inform readers of the above concerns, which cannot be fully resolved in the absence of the original underlying data, and recommend that readers consult the Supporting Information files provided with this notice when interpreting the results presented in Figs 4 and 5.

## Supporting information

S1 File Modified Fig 4, incorporating replacement panels obtained in replication experiments. (TIF)

S2 File Original data underlying the modified Fig 4. (PDF)

S3 File Individual-level data underlying published results. (XLSX)

S4 File PowerPoint file provided in support of Fig 5 results. (PPTX)
