# Supplementary figures and images for: Expression of Concern: Unliganded Estrogen Receptor Alpha Promotes PC12 Survival during Serum Starvation
Source: PLoS One. 2024 Jan 11;19(1):e0297382. doi: 10.1371/journal.pone.0297382 (PMC10783772; doi:10.1371/journal.pone.0297382)

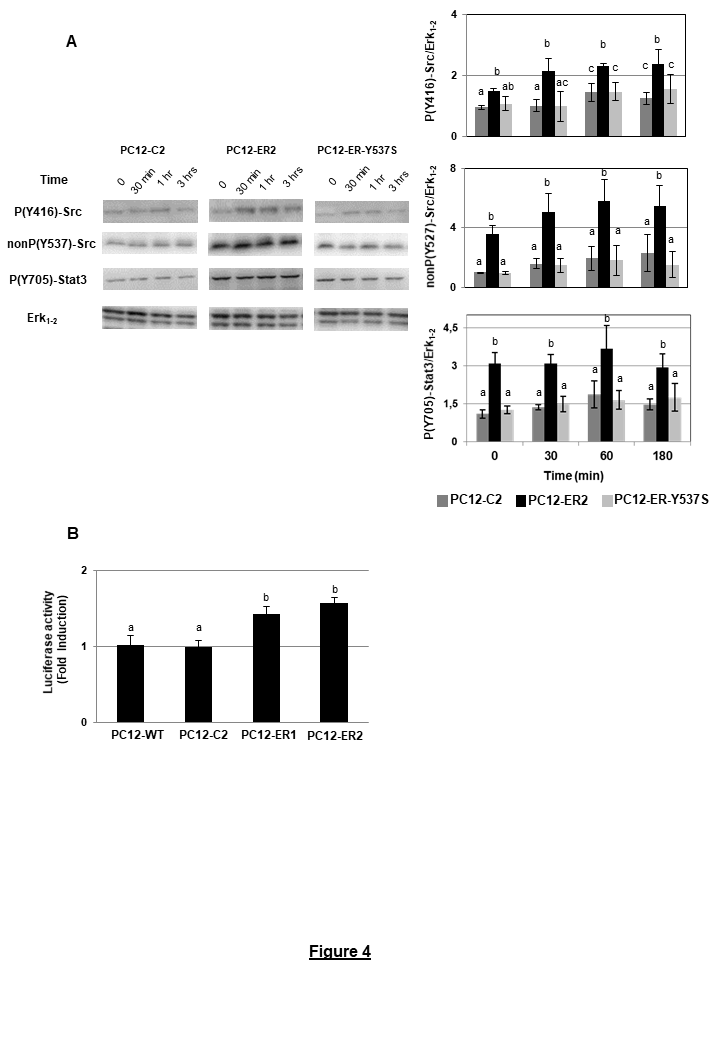

Supplement: S1 File — (TIF) [file pone.0297382.s001.tif]

## Erk1-2

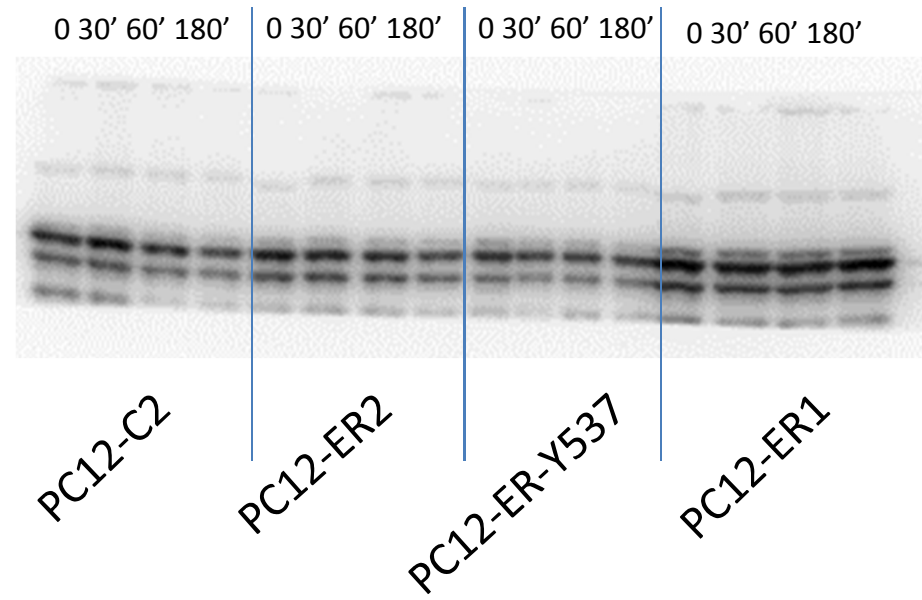

P(Y416)-Src

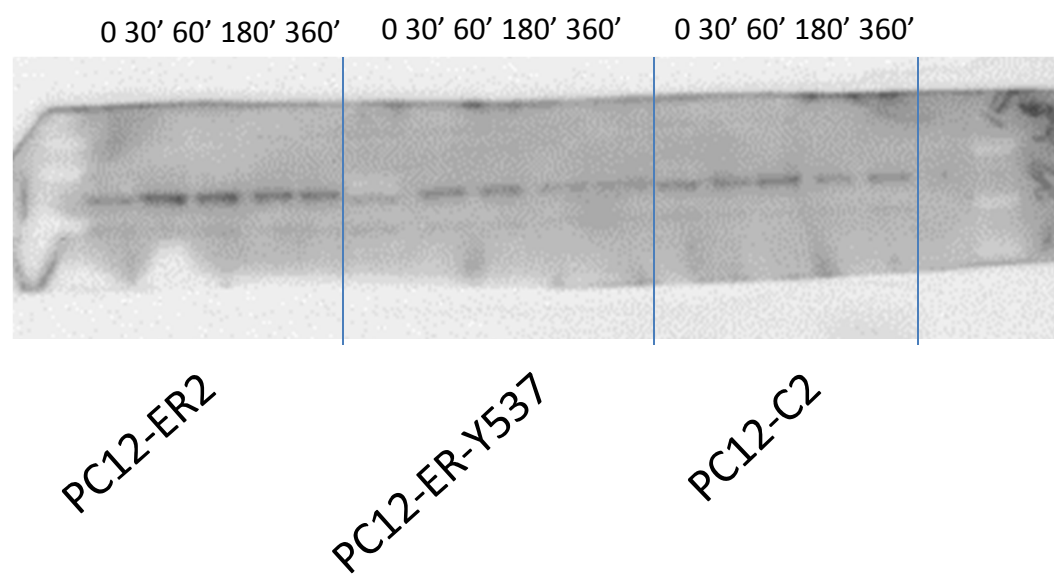

nonP(Y527)-Src

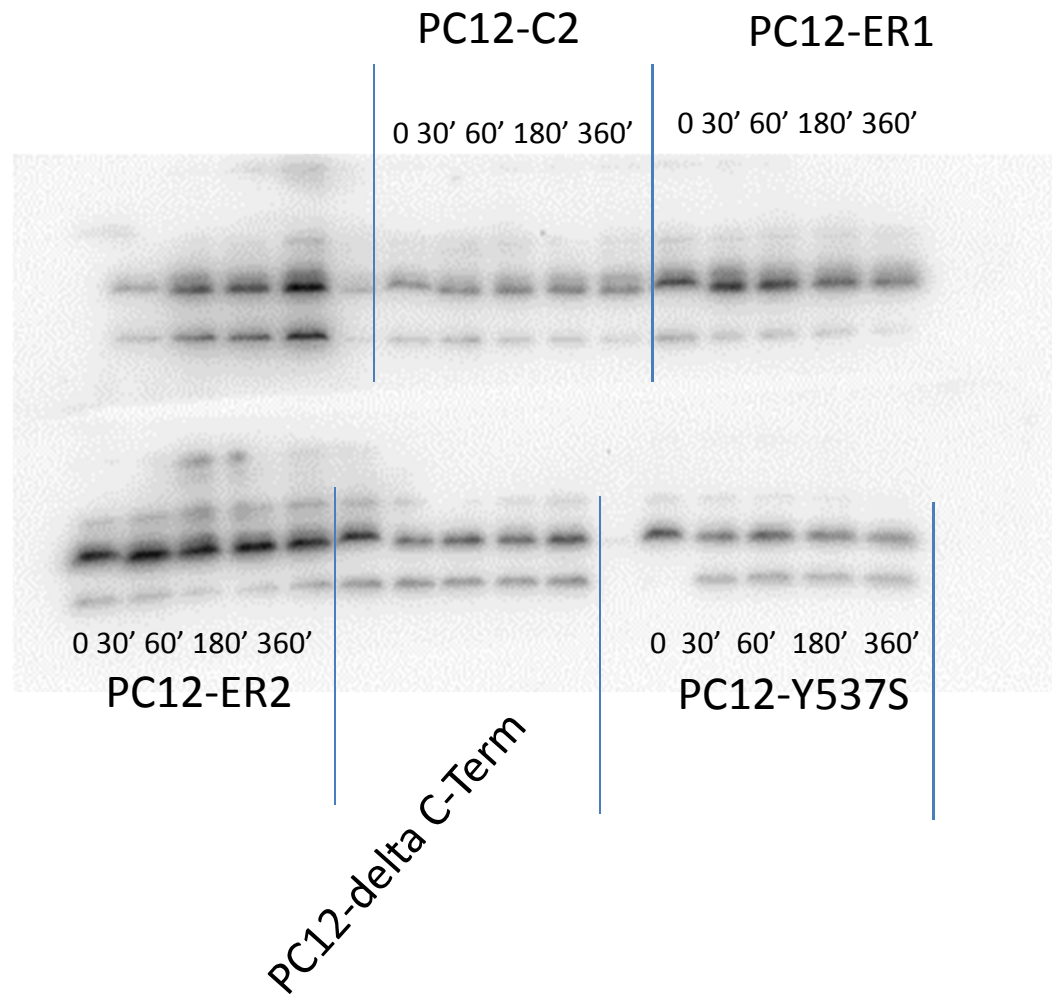

P(Y705)-Sta3

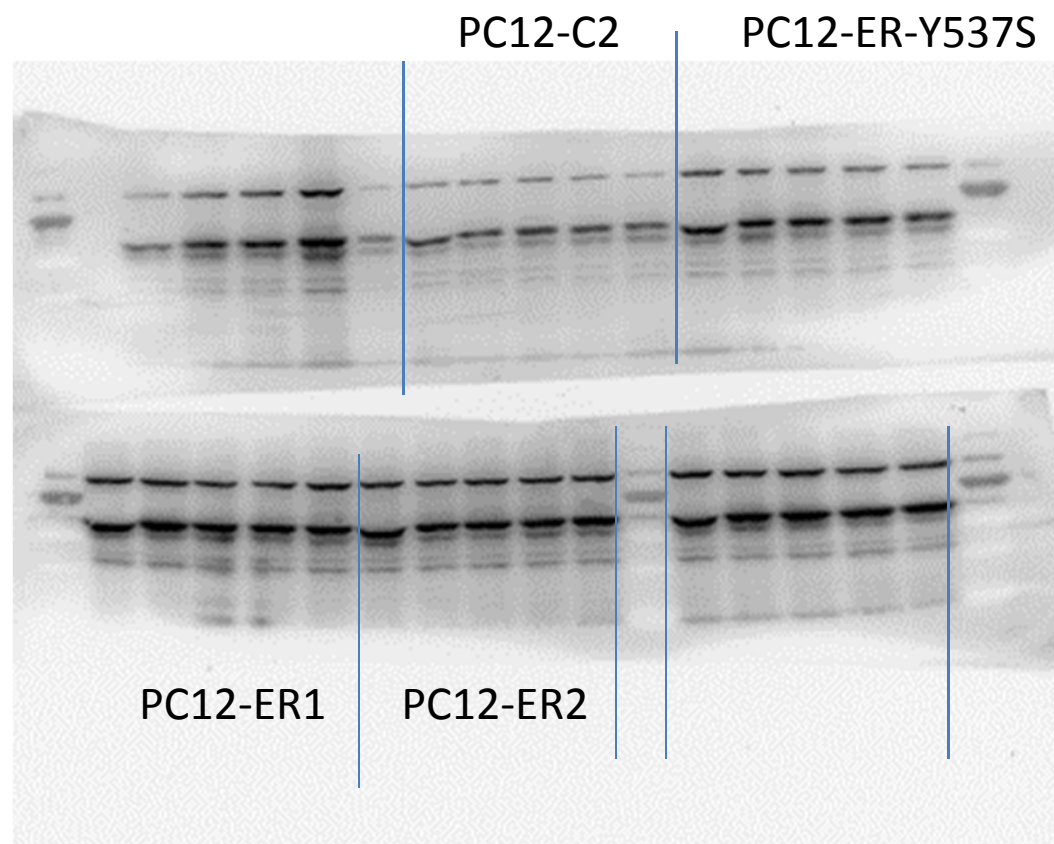

Supplement: S2 File — (PDF) [file pone.0297382.s002.pdf]

## Slide 1
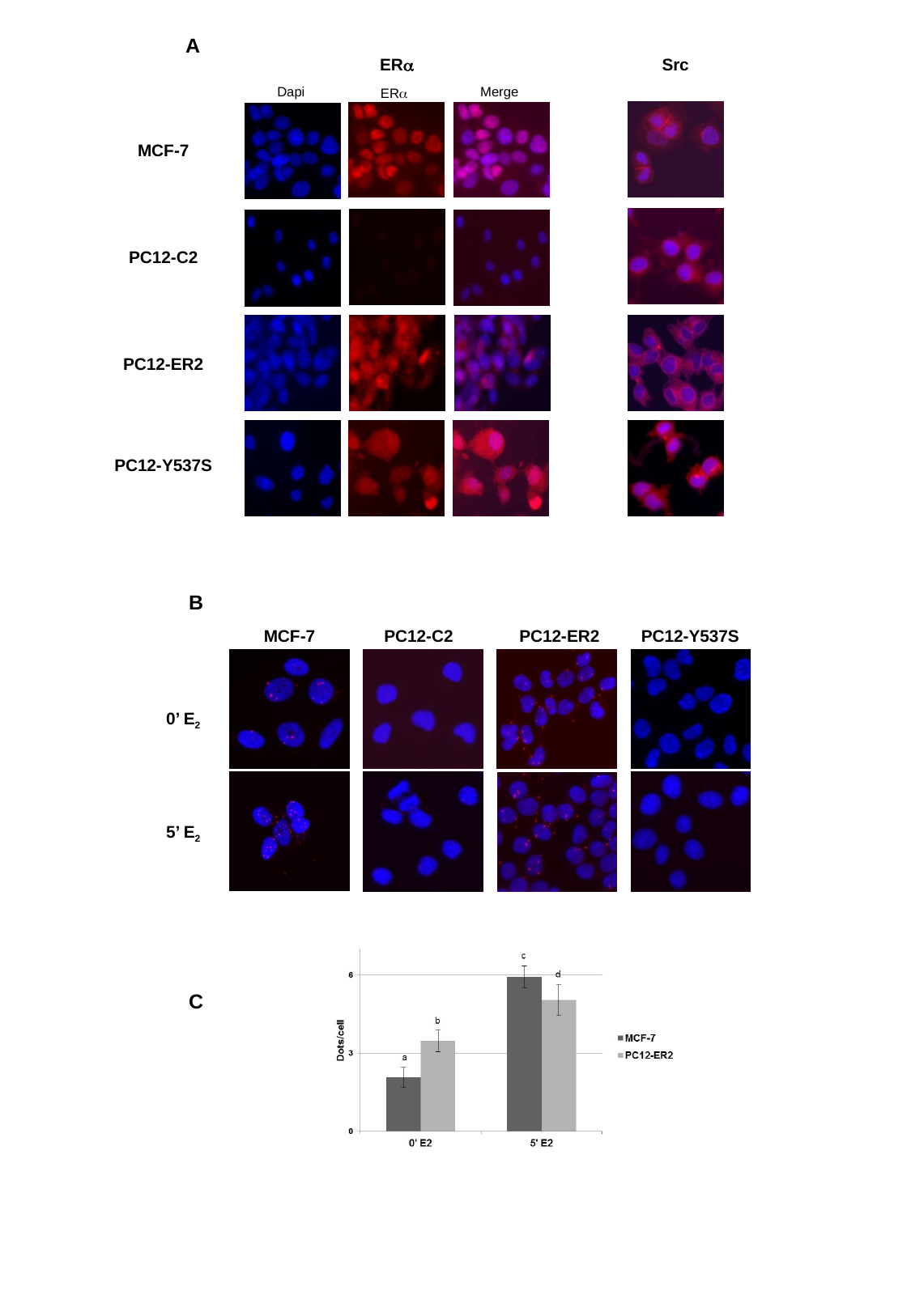

A
ERa
Src
MCF-7
PC12-C2
PC12-ER2
PC12-Y537S
Dapi
Merge
ERa
B
MCF-7
PC12-C2
PC12-ER2
PC12-Y537S
0’ E2
5’ E2
C

Supplement: S4 File — (PPTX) [file pone.0297382.s004.pptx]
